# Exploratory Antinociceptive and Anti‐Inflammatory Effects of *Dittrichia viscosa* Extracts: HPLC‐PDA Fingerprinting, Preliminary Acute Tolerability, ADMET Prediction, and Docking‐Based Prioritization

**DOI:** 10.1155/prm/7424265

**Published:** 2026-10-07

**Authors:** Sara Tlemcani, Oussama Khibech, Amal Lahkimi, Abdessadek Essadek, Chin-Hung Lai, Anouar Hmamou, Amine Mounadi Idrissi, Maryem Aichouch, Amal Elrherabi, Mohamed Bouhrim, Hicham Bekkari

**Affiliations:** ^1^ Laboratory of Biotechnology, Environment, Agri-Food, and Health Faculty of Sciences Dhar El Mahraz Sidi Mohamed Ben Abdellah University B.P. 1796 Atlas Fez 30000 Morocco usmba.ac.ma; ^2^ Laboratory of Engineering, Electrochemistry, Modeling and Environment Faculty of Sciences Dhar El Mahraz Sidi Mohamed Ben Abdellah University B.P. 1796 Atlas Fez 30000 Morocco usmba.ac.ma; ^3^ Laboratory of Applied Chemistry and Environment Faculty of Sciences Mohammed First University BP 717 60000 Oujda Morocco; ^4^ Laboratory of Bioresources, Biotechnology, and Sustainable Development, Faculty of Sciences, Mohammed First University, BV Mohammed VI BP 717, 60000, Oujda, Morocco; ^5^ Department of Medical Applied Chemistry Chung Shan Medical University 402 Taichung Taiwan csmu.edu.tw; ^6^ Department of Chemistry Laboratory of Engineering, Organometallic and Molecular Materials and Environment Faculty of Sciences Dhar El Mahraz Sidi Mohamed Ben Abdellah University Fez Morocco usmba.ac.ma; ^7^ Biological Engineering Laboratory Faculty of Sciences and Techniques Sultan Moulay Slimane University Beni Mellal Morocco universitesms.com

**Keywords:** antinociceptive activity, carrageenan, *Dittrichia viscosa*, exploratory animal study, formalin test, HPLC-PDA fingerprinting, inflammation, molecular docking

## Abstract

The present exploratory study examined a hydroethanolic extract (HEE) and an ethyl‐acetate‐derived fraction operationally termed the polyphenolic fraction (PF) of *Dittrichia viscosa* by combining HPLC‐PDA fingerprinting, acute nociception and inflammation models, a 72‐h acute oral tolerability screen of HEE, and in silico prioritization. The HPLC‐PDA fingerprint of HEE contained 31 peaks. Because the available analytical record did not support validated absolute quantification, platform‐exported values are reported only as relative chromatographic abundance. The larger peaks included p‐coumaric acid (7.20%), caffeic acid (5.11%), a putative caffeic acid‐O‐hexoside dimer (4.55%), a putative isorhamnetin‐O‐hexoside (3.57%), a putative hydroxybenzoic acid hexoside (3.25%), and apigenin (3.23%); six annotations were standard‐supported, whereas the remaining assignments were putative. In rats (*n* = 5/group), every treatment‐control comparison in the writhing and formalin models was significant in the summary‐statistic Dunnett reanalysis (adjusted *p* < 0.0001). PF at 100 mg/kg produced 59.5% writhing inhibition and mean differences versus vehicle of −38.46 s (95% CI −40.42 to −36.50) and −23.00 s (95% CI −24.45 to −21.55) in the early and late formalin phases, respectively. In the carrageenan model, significant differences occurred mainly at 6 h; PF at 100 mg/kg differed from vehicle by −0.24 cm (95% CI −0.34 to −0.14; adjusted *p* = 0.0002). Percentage inhibition values are descriptive. No mortality or overt clinical signs were observed after HEE doses up to 4000 mg/kg during 72 h, which constitutes preliminary acute tolerability evidence for HEE only. Predicted ADMET outputs and AutoDock Vina scores prioritized candidate ligands but do not establish exposure, safety, target inhibition, or causal contribution to the extract‐level effects. These exploratory findings support further preclinical investigation, subject to chemical confirmation, raw‐data‐based longitudinal statistics, direct target assays, and guideline‐compliant safety studies.

## 1. Introduction

Pain and inflammation involve interacting peripheral and central pathways activated by prostaglandins, nitric oxide, leukotrienes, bradykinin, cytokines, and reactive oxygen species. Although nonsteroidal anti‐inflammatory drugs, paracetamol, and opioids remain widely used, their effectiveness and tolerability limitations motivate the search for additional preclinical candidates [1–4]. Natural products remain relevant because phenolic acids and flavonoids can influence oxidative and inflammatory signaling in experimental systems, but the activity of a botanical extract cannot be assigned to individual compounds without isolation, authentication, and direct testing [5–10]. *Dittrichia viscosa* (L.) Greuter is a Mediterranean Asteraceae species used in traditional medicine and reported to contain phenolic acids, flavonoids, terpenoids, and other specialized metabolites [11–15]. TRPA1, COX‐2, 5‐LOX, and inducible nitric oxide synthase (iNOS) are biologically relevant to nociceptive and inflammatory signaling [16–20]; however, the present study did not directly measure these targets. They were therefore used only for hypothesis‐generating computational prioritization.

Accordingly, this exploratory study aimed to: (i) describe an HPLC‐PDA fingerprint of the Moroccan D. viscosa hydroethanolic extract (HEE) without claiming unsupported absolute quantification; (ii) evaluate HEE and polyphenolic fraction (PF) in acetic acid, formalin, and carrageenan models; (iii) report a preliminary 72‐h acute oral tolerability screen of HEE only; and (iv) prioritize a limited set of structurally defined candidate ligands by predicted ADMET descriptors and docking against iNOS, COX‐2, the AKBA‐defined allosteric site of 5‐LOX, and TRPA1. The pharmacological, analytical, and computational evidence levels were interpreted separately.

## 2. Materials and Methods

### 2.1. Plant Samples

Aerial plant material was collected in El Menzel Province, Sefrou, Fes‐Meknes, Morocco (33°50′11.99″ N, 4°32′26.99″ W), during March and April at the growth and flowering stages. The species was identified by botanist Eloutassi Noureddine, Regional Center for Education and Training, Fez, and assigned voucher number FMM/032102. The repository name and accession‐verification record were not retained in the documentation available for this revision; therefore, the previous unsupported statement concerning recognition by an external association was removed.

### 2.2. HEE Preparation

Twenty grams of washed plant powder were oven‐dried at 45°C for 72 h and macerated for 48 h in 70% ethanol/30% distilled water. The mixture was filtered through Whatman paper, and the solvent was removed under reduced pressure using a BUCHI R‐100 rotary evaporator (New Castle, DE, USA) [21]. The dried HEE was stored at 6°C in closed tubes until use, and the extraction yield was 22.8%. The available source record did not retain the particle‐size range, solvent volume, filter grade, exact number of hydroethanolic extraction cycles, storage duration, or stability assessment; these omissions are acknowledged as reproducibility limitations rather than reconstructed retrospectively.

### 2.3. Ethyl‐Acetate‐Derived Fraction Preparation

Ten grams of *D. viscosa* powder underwent three successive macerations with 30‐mL methanol per cycle. The pooled filtrates were concentrated at low temperatures, redissolved in 50‐mL distilled water, and extracted sequentially three times each with 20‐mL hexane, 20‐mL chloroform, and 20‐mL ethyl acetate. The ethyl acetate layer was collected and evaporated under vacuum at 40°C. The resulting fraction yielded 6.27% and is termed the PF throughout the manuscript for consistency. This is an operational extraction label; direct quantitative confirmation of polyphenol enrichment in PF was not available.

### 2.4. HPLC‐PDA Fingerprinting

The analytical record available for this revision attributes the chromatogram reported in Figure 1 to a Waters Alliance HPLC system comprising an e2695 separation module and a 2998 photodiode‐array detector (PDA), controlled with Empower 3 software. The unrelated preparative‐HPLC and Thermo/Accela instrumentation descriptions that appeared in the preceding version were removed because they did not generate the reported fingerprint. Reversed‐phase separation used a Kinetex C8 column (100 × 2.1 mm, 2.6 μm particles, 100 Å pore size) protected by a C8 Security Guard cartridge. The injection volume was 10 μL, the column temperature was 35°C, and the representative trace was reported at 254 nm.

**FIGURE 1:**
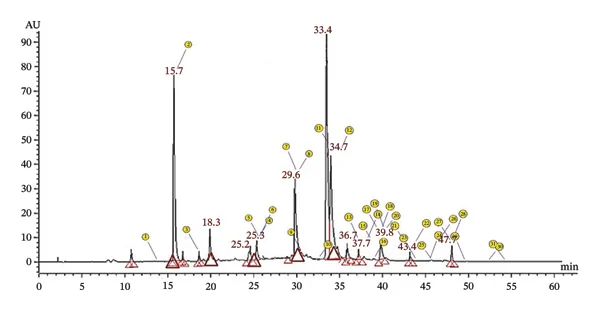
Representative HPLC‐PDA chromatographic profile of the *Dittrichia viscosa* extract recorded at 254 nm.

The complete archived mobile‐phase composition, gradient, flow rate, sample‐preparation record, injection‐replication record, calibration equations, linearity, LOD/LOQ, precision, accuracy, and recovery data were not available in the source package. Consequently, the HPLC‐PDA analysis is presented strictly as an exploratory fingerprint and not as a validated quantitative assay. No absolute concentration in mg/g is reported in the revised manuscript.

Peaks were numbered by retention‐time order and linked to Table 1. Caffeic acid, p‐coumaric acid, luteolin, apigenin, isorhamnetin, and quercetin dihydrate are reported as standard‐supported annotations because the available record states that retention time and PDA spectra were compared with authentic standards under the same analytical conditions. All other structural assignments are putative and are based on retention behavior, PDA spectral features, proposed formula, and literature comparison; LC‐MS/MS confirmation was not performed. The numeric values in Table 1 are the platform‐exported relative chromatographic abundances associated with integrated peaks. They are not mg/g concentrations, standard deviations, or method‐validation percentages. The HPLC‐PDA fingerprint characterizes HEE and does not establish the composition of PF.

**TABLE 1:** Peak assignments, retention times, molecular formulas, platform‐exported relative chromatographic abundance, and identification status in the *Dittrichia viscosa* HEE HPLC‐PDA fingerprint.

Peak no.	Compounds	tR (min)	Molecular formula	Relative chromatographic abundance (%)	Status
1	Caffeic acid‐O‐hexoside	13.6	—	< LoQ	Putative
2	Caffeic acid	15.7	C9H8O4	5.11	Std‐supported
3	p‐Coumaric acid	18.3	C9H8O3	7.20	Std‐supported
4	Taxifolin hexoside	25.2	C21H22O12	2.80	Putative
5	Hydroxybenzoic acid hexoside	25.3	C13H16O8	3.25	Putative
6	Isorhamnetin‐O‐hexoside	25.6	C22H22O12	3.57	Putative
7	3,4‐Dicaffeoylquinic acid	29.6	C22H24O12	2.59	Putative
8	3,5‐Dicaffeoylquinic acid	29.8	C22H24O12	1.87	Putative
9	4,5‐Dicaffeoylquinic acid	30.2	C22H24O12	2.18	Putative
10	Coumaryl caffeoylquinic acid	32.1	—	1.94	Putative
11	Caffeic acid‐O‐hexoside dimer	33.4	—	4.55	Putative
12	Luteolin	34.7	C15H10O6	2.71	Std‐supported
13	Isorhamnetin‐3‐O‐(6‐O‐feruloyl)‐glucoside	36.7	—	0.64	Putative
14	Hispidulin hexoside	37.7	C22H22O11	1.65	Putative
15	Patuletin	38.2	C16H12O8	1.68	Putative
16	7‐O‐Methylaromadendrin	38.5	C16H14O6	2.31	Putative
17	Padmatin	38.9	C16H14O7	0.32	Putative
18	3‐O‐Acetylaromadendrin	39.4	—	0.67	Putative
19	Spinacetin	39.7	C16H12O8	0.71	Putative
20	Apigenin	39.8	C15H10O5	3.23	Std‐supported
21	Hispidulin	42.1	C16H12O6	0.54	Putative
22	3,3′‐Di‐O‐methylquercetin	43.2	C17H14O7	1.35	Putative
23	3‐O‐Methylquercetin	43.4	C16H12O7	2.33	Putative
24	Rhamnocitrin	44.9	C16H12O6	0.64	Putative
25	Isorhamnetin	45.2	C16H12O7	0.71	Std‐supported
26	Helenin	46.3	C15H20O2	—	Unres.
27	Quercetin dihydrate	47.4	C15H14O9	1.58	Std‐supported
28	Nepetin	47.7	C16H12O7	12.71	Putative
29	3‐O‐Acetylpadmatin	49.0	C18H16O8	0.22	Putative
30	Sakuranetin	51.6	C16H14O5	0.84	Putative
31	Genkwanin	53.7	C16H12O5	1.36	Putative

*Note:* tR, retention time; Std, standard‐supported by retention‐time and PDA‐spectrum comparison with an authentic standard; Put, putative assignment from retention behavior, PDA features, proposed formula, and literature comparison; Unres., unresolved annotation. Relative chromatographic abundance values are integrated peak‐area percentages exported with the available peak table; they are not mg/g concentrations, SDs, or validation statistics. The legacy label < LoQ is reproduced from the platform export, but the underlying validated LoQ record was unavailable. LC‐MS/MS confirmation and a validated quantitative method were not available.

Abbreviation: HEE, hydroethanolic extract.

### 2.5. Animals and Reporting of Experimental Design

Male Wistar rats weighing 100–150 g were used in the antinociceptive and anti‐inflammatory experiments. Swiss albino mice weighing 25–30 g were used for the preliminary HEE tolerability screen. Animals were housed at 22 ± 2°C and 50%–55% relative humidity under a 12‐h light/dark cycle, with free access to food and water. Procedures followed institutional standards for laboratory‐animal care [22] and the ethics approval reported in the Declarations.

Five rats per group (*n* = 5) were selected before experimentation by reference to previous plant‐extract studies using writhing, formalin, and carrageenan models [23–25] and to the 3Rs principle. No prospective power calculation was performed. The sample size was therefore intended to detect large signals in an exploratory study, not to provide definitive efficacy estimates. Results are reported with mean differences, 95% confidence intervals where estimable, and explicit small‐sample limitations.

### 2.6. Allocation, Blinding, and Treatment Preparation

The study records described group allocation as random but did not retain the sequence‐generation method, allocation‐concealment procedure, or outcome‐assessor blinding status. The negative‐control vehicle was 0.9% NaCl; however, the available records did not preserve the final preparation concentration, suspension procedure, additives, or administration volume for each HEE and PF dose. No undocumented procedural details were reconstructed retrospectively. These reporting gaps are stated explicitly because the behavioral endpoints may be susceptible to allocation and observation bias.

### 2.7. Acute Antinociceptive Activity In Vivo

#### 2.7.1. Acetic Acid–Induced Writhing

The acetic acid–induced writhing model was conducted according to Konaté et al. (2012) [23]. Thirty rats were allocated to six groups (*n* = 5): vehicle control (0.9% NaCl, 10 mL/kg), paracetamol (10 mg/kg), HEE (200 or 400 mg/kg), or PF (50 or 100 mg/kg). Treatments were administered orally. One hour later, 1% acetic acid was injected intraperitoneally at 10 mL/kg. After a 5‐min latency, abdominal constrictions were counted for 30 min. This assay provides evidence of an antinociceptive response in a chemically induced model but does not identify a specific molecular target.

#### 2.7.2. Formalin Test

The formalin test was conducted according to Coderre et al. [25]. Thirty rats were allocated to vehicle control, paracetamol (10 mg/kg), HEE (200 or 400 mg/kg), or PF (50 or 100 mg/kg) groups (*n* = 5). Thirty minutes after treatment, 20 μL of 2% formalin was injected subcutaneously into the plantar surface of the right hind paw. The total time spent licking or biting the injected paw was recorded during the early phase (0–5 min) and late phase (15–30 min). Reduction of either phase is interpreted as antinociceptive activity in this model and not as evidence of direct TRPA1 or other target engagement.

### 2.8. Carrageenan‐Induced Paw Edema

The carrageenan experiment followed the cited plant‐extract protocols [24, 26]. After a 12‐h fast, 30 rats were allocated to vehicle control, diclofenac (15 mg/kg), HEE (200 or 400 mg/kg), or PF (50 or 100 mg/kg) groups (*n* = 5). One hour after oral treatment, 5 μL of 1% carrageenan was injected subcutaneously into the plantar surface of the right hind paw. The same animals were measured before injection (*V*_0_) and at 3, 4, 5, and 6 h. The archived table reports paw diameter in centimeters; the measurement‐instrument model and assessor‐blinding status were not retained in the available study record and are therefore disclosed as methodological limitations.

Edema inhibition (EI) was calculated descriptively from baseline‐corrected group means:

(1) EI%=ΔVc−ΔVtΔVc×100,

where Δ*V*_*c*_ is the control‐group change from baseline and Δ*V*_*t*_ is the treated‐group change. Statistical inference was based on the reported paw measurements rather than on EI percentages.

### 2.9. Preliminary Acute Oral Tolerability Test

Only HEE was evaluated in the acute oral tolerability experiment. The procedure was adapted from Hilan et al. [27] and was not a complete OECD 423 or OECD 425 study. HEE was diluted in 0.9% NaCl and administered orally to mice at 600, 1000, 2000, or 4000 mg/kg; a fifth group received vehicle only (*n* = 6/group). Animals were observed during the immediate post‐dose period and for 72 h for mortality and visible clinical signs, including changes in activity, posture, grooming, piloerection, salivation, tremor, convulsions, diarrhea, respiration, lethargy, and coma. The experiment did not assess PF, 14‐day body‐weight change, hematology, clinical chemistry, or histopathology and cannot establish a general safety profile or regulatory LD50 classification.

### 2.10. Statistical Analysis

Values are reported as mean ± SD from biological replicates (individual animals), unless otherwise specified. Inhibition percentages were calculated from group means and are descriptive. For the writhing and formalin endpoints, one‐way ANOVA was followed by Dunnett‐adjusted comparisons of each active‐treatment group with its corresponding vehicle control. Carrageenan measurements were obtained repeatedly from the same animals. Because individual longitudinal records and within‐animal covariance were not available for the revision, a mixed‐effects or repeated‐measures model could not be reconstructed. Separate, exploratory one‐way ANOVAs with Dunnett adjustment were therefore recalculated at each time point; these tests do not estimate a group‐by‐time interaction. The revision reanalysis used the reported rounded group means, SDs, and *n* values in SciPy 1.17.0, so adjusted *p*‐values are approximate. Welch 95% confidence intervals were calculated for selected treatment‐control mean differences. For carrageenan, EI (%) = [(Δ*V*_*c*_ − Δ*V*_*t*_)/Δ*V*_*c*_] × 100. A two‐sided adjusted *p* < 0.05 was considered statistically significant.

### 2.11. In Silico ADMET Prediction

Six structurally defined compounds were selected before docking using explicit criteria: (i) an unambiguous chemical structure suitable for computation; (ii) either a standard‐supported HPLC‐PDA annotation (caffeic acid, p‐coumaric acid, luteolin, and apigenin) or a literature‐supported putative *Dittrichia/Inula* flavonoid (nepetin and 3‐O‐methylquercetin); (iii) representation of phenolic acid and flavonoid scaffolds; and (iv) literature relevance to inflammatory or nociceptive pathways. Putative high‐abundance annotations such as 3‐O‐acetylpadmatin, hispidulin, and padmatin were not included because the available data did not provide sufficiently secure structural confirmation for compound‐specific modeling. Canonical structures were submitted to SwissADME, pkCSM, and ADMET‐AI [28–32]. All outputs are model predictions for isolated compounds and do not represent measured pharmacokinetics, toxicity, or composition of the administered HEE/PF.

### 2.12. Molecular Docking

Docking was performed with AutoDock Vina v1.2.0 in PyRx v0.9 [33–35]. The structures were human iNOS (PDB 4NOS; pocket defined by ethylisothiourea, ITU), human COX‐2 (5KIR; rofecoxib, RCX), human 5‐LOX (6NCF; AKBA, AF7), and human TRPA1 (6WJ5; GDC‐0334, LXY). The 6NCF grid corresponds to the experimentally observed AKBA allosteric pocket rather than the catalytic iron site. Receptors were prepared in AutoDockTools v1.5.7 by removing crystallographic waters and nonessential heteroatoms, adding polar hydrogens, assigning charges, and converting to PDBQT. Receptors were treated as rigid and ligand torsions as flexible. Explicit ligand microstate/tautomer enumeration, flexible receptor residues, and a complete retrospective cofactor/metal‐retention audit were not available in the archived workflow and are acknowledged as limitations. The native ligand was removed and redocked with exhaustiveness 10. Grid centers/sizes (Angstrom) and pose‐recovery RMSDs were: 4NOS, center (−1.90, 97.95, 20.97), size (14.58, 12.47, 14.52), RMSD 1.116 Å; 5KIR, center (23.24, 0.50, 34.52), size (12.82, 14.52, 14.98), RMSD 1.337 Å; 6NCF, center (11.34, −22.32, −18.33), size (18.21, 7.39, 9.94), RMSD 0.174 Å; and 6WJ5, center (156.96, 140.22, 185.17), size (15.46, 11.75, 14.30), RMSD 0.818 Å. RMSD below 2 Å supports recovery of the crystallographic pose but does not validate score‐to‐affinity accuracy. The top‐ranked predicted pose was mapped in PyMOL v2.5 and BIOVIA Discovery Studio 2024. Vina scores are empirical predicted docking scores, not experimental binding energies, residence times, stability measurements, or evidence of target inhibition.

## 3. Results

### 3.1. HPLC‐PDA Fingerprint of the HEE

The representative HPLC‐PDA trace of HEE at 254 nm contained 31 numbered peaks linked to Table 1 (Figure 1). Platform‐exported relative chromatographic abundance was greatest for p‐coumaric acid (7.20%), caffeic acid (5.11%), a putative caffeic acid‐O‐hexoside dimer (4.55%), a putative isorhamnetin‐O‐hexoside (3.57%), a putative hydroxybenzoic acid hexoside (3.25%), and apigenin (3.23%). Six peaks are standard‐supported; the remaining annotations are putative. The values describe a single chromatographic fingerprint and are not validated mg/g concentrations, uncertainty estimates, or evidence that the listed compounds occur in PF.

### 3.2. Exploratory Antinociceptive Activity In Vivo

#### 3.2.1. Acetic Acid–Induced Writhing

All active treatments reduced writhing counts versus vehicle in the summary‐statistic Dunnett reanalysis (adjusted *p* < 0.0001 for each comparison; Table 2). Mean counts were 70.20 ± 2.97 in the control, 47.20 ± 2.92 and 30.20 ± 1.85 with HEE at 200 and 400 mg/kg, and 32.80 ± 0.83 and 28.40 ± 1.67 with PF at 50 and 100 mg/kg, respectively.

**TABLE 2:** Acetic acid–induced writhing in rats (*n* = 5 biological replicates/group).

Treatments	Writhing count (mean ± SD)	Inhibition (%)
Vehicle control (0.9% NaCl)	70.20 ± 2.97	0.0
Paracetamol (10 mg/kg)	20.20 ± 2.52	71.2
HEE (200 mg/kg)	47.20 ± 2.92	32.8
HEE (400 mg/kg)	30.20 ± 1.85	57.0
PF (50 mg/kg)	32.80 ± 0.83	53.3
PF (100 mg/kg)	28.40 ± 1.67	59.5

*Note:* Values are mean ± SD. Every active‐treatment versus vehicle comparison had Dunnett‐adjusted *p* < 0.0001. Inhibition percentages were calculated descriptively from group means.

The corresponding descriptive inhibitions were 32.8% and 57.0% for HEE and 53.3% and 59.5% for PF. For PF at 100 mg/kg, the treatment‐control mean difference was −41.80 writhes (95% CI −45.49 to −38.11). The higher tested dose within each preparation produced a larger numerical inhibition, but two doses do not establish a formal dose–response relationship. Because HEE and PF were tested at nonequivalent dose ranges and were not chemically standardized, these data do not establish greater intrinsic potency of PF.

#### 3.2.2. Formalin Test

Every active‐treatment versus vehicle comparison was significant in both formalin phases (Dunnett‐adjusted *p* < 0.0001; Table 3). PF at 100 mg/kg reduced the early‐phase mean from 56.00 ± 1.58 to 17.54 ± 0.16 s (mean difference −38.46 s, 95% CI −40.42 to −36.50) and the late‐phase mean from 31.20 ± 0.83 to 8.20 ± 1.11 s (mean difference −23.00 s, 95% CI −24.45 to −21.55). The descriptive inhibitions were 68.7% and 73.7%, respectively. These results demonstrate antinociceptive activity in the formalin model but do not identify TRPA1 or another molecular mechanism.

**TABLE 3:** Formalin‐induced licking/biting time in rats (*n* = 5 biological replicates/group).

Treatments	Early phase (0–5 min, s; mean ± SD)	Early inhibition (%)	Late phase (15–30 min, s; mean ± SD)	Late inhibition (%)
Vehicle control	56.00 ± 1.58	0.0	31.20 ± 0.83	0.0
Paracetamol (10 mg/kg)	9.49 ± 1.34	83.1	4.00 ± 0.57	87.2
HEE (200 mg/kg)	29.34 ± 0.75	47.6	16.15 ± 0.8	48.2
HEE (400 mg/kg)	25.86 ± 1.12	53.8	13.76 ± 0.75	55.9
Poly (50 mg/kg)	22.83 ± 1.10	59.2	11.13 ± 0.85	64.3
Poly (100 mg/kg)	17.54 ± 0.16	68.7	8.20 ± 1.11	73.7

*Note:* Values are mean ± SD. Every active‐treatment versus vehicle comparison had Dunnett‐adjusted *p* < 0.0001 in both phases. Inhibition percentages were calculated descriptively from group means.

### 3.3. Exploratory Anti‐Inflammatory Activity

Carrageenan measurements and descriptive edema‐inhibition percentages are shown in Table 4. No treatment‐control comparison was statistically significant at 3 or 4 h in the summary‐statistic Dunnett reanalysis. At 5 h, only PF at 100 mg/kg differed from vehicle (adjusted *p* = 0.0078). At 6 h, diclofenac, both HEE doses, and both PF doses differed from vehicle (all adjusted *p* ≤ 0.0040). PF at 100 mg/kg had a 6‐h mean difference of −0.24 cm (95% CI −0.34 to −0.14; adjusted *p* = 0.0002). The corresponding 77.5% EI value is descriptive and does not itself provide a significance test. Because the same animals were measured repeatedly and only summary data were available, these separate time‐point tests do not establish a longitudinal group‐by‐time effect.

**TABLE 4:** Carrageenan‐induced paw measurements in rats (*n* = 5 biological replicates/group).

Treatments	V0 (cm)	T3 (cm; EI %)	T4 (cm; EI %)	T5 (cm; EI %)	T6 (cm; EI %)
Vehicle control	2.02 ± 0.083	2.34 ± 0.09 (0.0)	2.50 ± 0.95 (0.0)	2.44 ± 0.09 (0.0)	2.42 ± 0.04 (0.0)
Diclofenac (15 mg/kg)	2.12 ± 0.13	2.33 ± 0.083 (34.4)	2.31 ± 0.05 (60.4)	2.26 ± 0.05 (66.7)	2.21 ± 0.08^∗∗∗^ (77.5)
HEE (200 mg/kg)	2.10 ± 0.10	2.32 ± 0.16 (31.3)	2.32 ± 0.06 (54.2)	2.27 ± 0.18 (59.5)	2.24 ± 0.05^∗∗^ (65.0)
HEE (400 mg/kg)	2.07 ± 0.21	2.28 ± 0.19 (34.4)	2.34 ± 0.22 (43.8)	2.27 ± 0.07 (52.4)	2.20 ± 0.11^∗∗∗^ (67.5)
Poly (50 mg/kg)	2.12 ± 0.05	2.31 ± 0.11 (40.6)	2.33 ± 0.09 (56.3)	2.27 ± 0.10 (64.3)	2.23 ± 0.07^∗∗^ (72.5)
Poly (100 mg/kg)	2.09 ± 0.18	2.30 ± 0.21 (34.4)	2.28 ± 0.26 (60.4)	2.20 ± 0.11^∗∗^ (73.8)	2.18 ± 0.08^∗∗∗^ (77.5)

*Note:* Values are mean ± SD; EI (%) in parentheses was calculated descriptively from baseline‐corrected group means. Dunnett‐adjusted comparisons are against vehicle at the same time point.

^∗∗^ *p* < 0.01.

^∗∗∗^ *p* < 0.001.

Approximate Dunnett‐adjusted *p*‐values (derived from the reported rounded means, SDs, and *n*) were as follows. At T3: diclofenac 1.0000, HEE 200 mg/kg 0.9997, HEE 400 mg/kg 0.9509, PF 50 mg/kg 0.9976, and PF 100 mg/kg 0.9910. At T4: 0.9207, 0.9350, 0.9587, 0.9477, and 0.8684, respectively. At T5: 0.0569, 0.0772, 0.0773, 0.0772, and 0.0078. At T6: 0.0008, 0.0040, 0.0005, 0.0023, and 0.0002. Baseline V0 comparisons were nonsignificant (all adjusted *p* ≥ 0.6720).

### 3.4. Preliminary Acute Oral Tolerability of HEE

No mortality or overt visible clinical signs were recorded in mice receiving HEE at 600–4000 mg/kg during the 72‐h observation period. This finding applies only to HEE and only to the endpoints and observation window used. PF was not tested. The result is therefore described as preliminary acute tolerability/absence of observed overt acute toxicity under the tested conditions and not as evidence of general safety, an LD50 category, or chronic tolerability.

### 3.5. In Silico ADMET Prioritization

The SwissADME bioavailability radar (Figure 2) summarizes predicted physicochemical properties of the six selected isolated compounds. The descriptors place most values within or near the platform‐defined drug‐likeness space, with recurrent low sp3 character typical of aromatic phenolics. These outputs are used only to compare computational profiles; they do not demonstrate oral bioavailability, systemic exposure, tissue concentrations, or the pharmacokinetics of HEE or PF.

**FIGURE 2:**
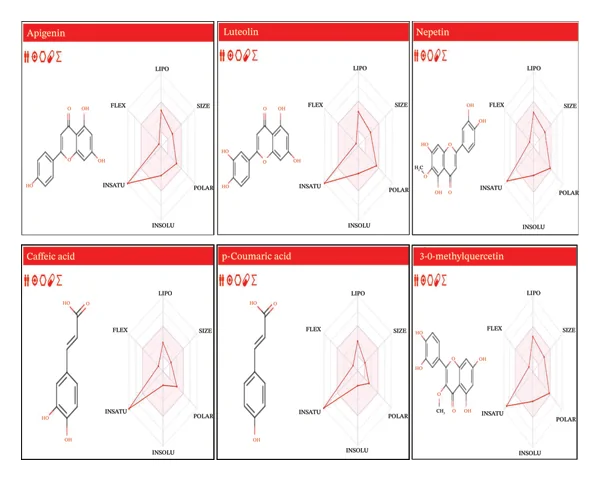
SwissADME bioavailability radar plots of the selected *Dittrichia viscosa* phytoconstituents.

Table 5 reports the SwissADME and pkCSM model outputs. All six structures had zero predicted Lipinski and Veber violations and a predicted high gastrointestinal‐absorption class, while quantitative absorption, distribution, CYP, transporter, and clearance values remained model estimates. Predicted low CNS permeability is compatible with, but does not establish, a predominantly peripheral mechanism. Similarly, predicted noninhibition or nonsubstrate classifications do not establish actual metabolic behavior or drug‐interaction safety. The ADMET‐AI map (Figure 3) placed the six structures within a comparatively low predicted clinical‐toxicity region of the platform reference space. This is a model‐based prioritization signal only; it does not demonstrate acute or chronic safety, does not apply directly to HEE or PF, and was not used to exclude experimental toxicological evaluation.

**TABLE 5:** Predicted ADME and physicochemical descriptors for selected *Dittrichia viscosa* candidate ligands.

Descriptor/predicted output	Apigenin	Luteolin	Nepetin	Caffeic acid	p‐Coumaric acid	3‐O‐methylquercetin
*Physicochemical properties*						
Molecular weight (g/mol)	270.24	286.24	316.26	180.16	164.16	316.26
H‐bond acceptors (count)	5	6	7	4	3	7
H‐bond donors (count)	3	4	4	3	2	4
Rotatable bonds (count)	1	1	2	2	2	2
Molar refractivity (cm^3^/mol)	73.99	76.01	82.5	47.16	45.13	82.5
TPSA (Å^2^)	90.9	111.13	120.36	77.76	57.53	120.36
Consensus Log P (o/w)	2.11	1.73	1.74	0.93	1.26	1.75
Predicted Log S (log mol/L)	−4.4	−3.82	−3.94	−0.71	−1.28	−3.94

*Drug-likeness and predicted oral-disposition descriptors*						
GI absorption (predicted class)	High	High	High	High	High	High
Lipinski violations (count)	0	0	0	0	0	0
Veber violations (count)	0	0	0	0	0	0
Bioavailability score (predicted)	0.55	0.55	0.55	0.56	0.85	0.55
Human intestinal absorption (predicted %)	93.25	81.13	77.294	69.407	93.494	76.069

*Predicted CNS distribution*						
BBB permeability (predicted log BB)	−0.734	−0.907	−1.378	−0.647	−0.225	−1.16
CNS permeability (predicted log PS)	−2.061	−2.251	−3.311	−2.608	−2.418	−3.268

*Predicted metabolism and transporter classifications*						
CYP2D6 inhibitor (predicted)	No	No	No	No	No	No
CYP3A4 inhibitor (predicted)	No	No	No	No	No	No
CYP2D6 substrate (predicted)	No	No	No	No	No	No
CYP3A4 substrate (predicted)	No	No	No	No	No	No

*Predicted excretion*						
Renal OCT2 substrate (predicted)	No	No	No	No	No	No
Total clearance (predicted log mL/min/kg)	0.566	0.495	0.463	0.508	0.662	0.457

*Note:* Values are in silico model outputs for isolated compounds and are not measured pharmacokinetic or safety data for HEE or PF.

**FIGURE 3:**
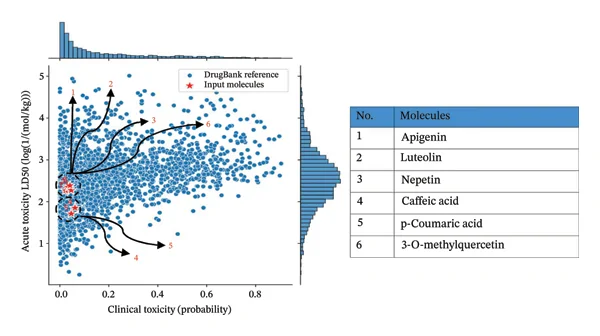
ADMET‐AI predicted toxicity landscape of the six selected candidate ligands relative to the DrugBank reference space; the adjacent key identifies the input molecules.

### 3.6. Molecular Docking Prioritization

Table 6 reports AutoDock Vina predicted docking scores. The more favorable phytochemical scores were luteolin for 4NOS (−9.9 kcal/mol), apigenin for 5KIR (−9.9 kcal/mol), nepetin at the AKBA‐defined 6NCF allosteric site (−7.9 kcal/mol), and 3‐O‐methylquercetin for 6WJ5 (−7.7 kcal/mol). These numerical rankings identify candidate ligands for subsequent testing; they are not experimental affinities, inhibition constants, residence times, or evidence that the compounds caused the in vivo extract‐level effects. Similarity to a co‐crystallized‐ligand score does not imply pharmacological equivalence. Figure 4 is retained as a descriptive pose map, while detailed claims of stable residence, strong affinity, or validated mechanism were removed.

**TABLE 6:** AutoDock Vina predicted docking scores (kcal/mol) of selected *Dittrichia viscosa* candidate ligands at iNOS (4NOS), COX‐2 (5KIR), the AKBA‐defined allosteric site of 5‐LOX (6NCF), and TRPA1 (6WJ5).

AutoDock Vina predicted docking score (kcal/mol)				
Candidate ligand	4NOS	5KIR	6NCF^∗^	6WJ5
Apigenin	−9.3	−9.9	−7.5	−7.6
Luteolin	−9.9	−9.6	−7.7	−7.6
Nepetin	−9.2	−8.8	−7.9	−7.4
Caffeic acid	−7.7	−7.3	−5.9	−6.5
p‐Coumaric acid	−7.3	−6.9	−5.4	−6.5
3‐O‐methylquercetin	−8.8	−9.5	−7.7	−7.7
Ethylisothiourea (ITU)	−4.4	N/A	N/A	N/A
Rofecoxib (RCX)	N/A	−10.0	N/A	N/A
AKBA (AF7)	N/A	N/A	−9.5	N/A
GDC‐0334 (LXY)	N/A	N/A	N/A	−10.1

*Note:* Scores are empirical model outputs, not measured binding energies or affinities. N/A, not applicable. Co‐crystallized ligands: ITU, ethylisothiourea (4NOS); RCX, rofecoxib (5KIR); AF7, AKBA (6NCF allosteric site); LXY, GDC‐0334 (6WJ5).

**FIGURE 4:**
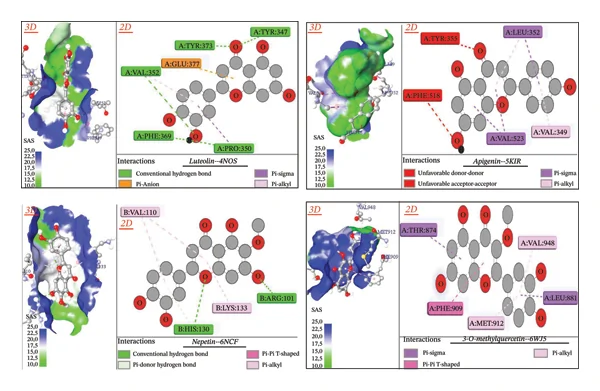
Two‐dimensional interaction maps and three‐dimensional solvent‐accessible surface representations of the top‐ranked predicted phytochemical poses: luteolin‐4NOS, apigenin‐5KIR, nepetin‐6NCF (AKBA‐defined allosteric site), and 3‐O‐methylquercetin‐6WJ5.

## 4. Discussion

The experimentally demonstrated findings are confined to the effects of the HEE and PF in acute rodent models and to an HPLC‐PDA fingerprint of HEE. The revised analytical section no longer reports unsupported absolute concentrations; instead, it presents relative peak‐area abundance and distinguishes six standard‐supported annotations from putative assignments. Because the fingerprint was generated for HEE, whereas PF was the lower‐dose preparation tested pharmacologically, the chemical composition and standardization of PF remain undetermined. The HEE profile is compatible with the phenolic and flavonoid diversity previously reported for *D. viscosa*, but it provides phytochemical context rather than a compound‐level explanation for PF activity [11, 13, 36].

In the acetic acid–induced writhing and formalin experiments [23, 25], all active treatments differed from vehicle in the summary‐statistic reanalysis, with large treatment‐control mean differences. These findings support exploratory antinociceptive activity in the tested models. However, the use of only two doses per preparation does not establish a formal dose–response relationship. Moreover, the nonequivalent dose ranges used for HEE and PF cannot establish greater intrinsic potency of PF because extraction yield, chemical composition, constituent exposure, and pharmacokinetics were not normalized. Reduction of both phases of the formalin response demonstrates activity within that model but does not identify TRPA1 or any other molecular target.

The carrageenan experiment [26, 37] provides exploratory evidence of a late anti‐inflammatory response. Statistical separation from vehicle was concentrated at 6 h, whereas the earlier time points were not significant in the available summary‐statistic reanalysis. Edema‐inhibition percentages describe the numerical magnitude of the response but should not be interpreted as evidence of a specific anti‐inflammatory mechanism or as a substitute for inferential statistics. Because the same animals were assessed repeatedly and individual longitudinal records were unavailable, a valid repeated‐measures or mixed‐effects model could not be reconstructed. Accordingly, no definitive group‐by‐time trajectory is claimed.

These observations are broadly consistent with previous reports of analgesic and anti‐inflammatory activity from plant‐derived preparations in rodent models, including *Capsicum annuum* and *Cicer arietinum* [38, 39]. Direct comparison of effect magnitude across studies is nevertheless inappropriate because plant species, plant material, extraction procedures, chemical standardization, administered doses, experimental designs, and outcome definitions differ. The present findings should therefore be regarded as a study‐specific preclinical signal rather than evidence of equivalence with other botanical preparations or reference drugs.

The tolerability observation requires similarly narrow interpretation. No mortality or overt clinical signs were observed during the 72‐h period following HEE administration, but PF was not evaluated toxicologically. OECD acute oral toxicity guidance generally includes a 14‐day observation period, repeated clinical observations, body‐weight monitoring, and gross necropsy [40, 41]. The present finding therefore supports only an absence of observed overt acute toxicity for HEE under the tested conditions; it cannot establish a broader safety profile or the safety of PF. Dedicated investigations would be required to evaluate hematological and biochemical parameters, histopathology, target‐organ toxicity, and repeated‐dose tolerability.

The computational layer is one step further removed from the pharmacological evidence. SwissADME, pkCSM, and ADMET‐AI provide predictions for isolated chemical structures [28, 29, 32] and cannot establish actual oral exposure, bioavailability, metabolic stability, CNS exclusion, CYP behavior, or safety of the administered botanical preparations. Similarly, AutoDock Vina scores are empirical ranking outputs rather than experimentally measured binding energies, affinities, or inhibitory activities. Successful redocking supports recovery of a crystallographic pose but does not independently validate scoring accuracy [33, 42, 43]. For 6NCF, the evaluated pocket corresponds to the AKBA‐defined allosteric site located between the membrane‐binding and catalytic domains of 5‐LOX [44]. The docking rankings therefore identify candidate ligand–pocket compatibility but do not demonstrate target inhibition, causal contribution to the in vivo effects, or the presence of any candidate in PF at a pharmacologically active concentration.

Accordingly, the integrated evidence is interpreted at three levels. Observed evidence consists of reductions in behavioral and edema‐related readouts and the absence of overt toxicity during the 72‐h HEE observation period. Supported inference is limited to possible involvement of inflammatory and nociceptive processes represented by the experimental models. Computational hypotheses concern the compatibility of selected candidate ligands with specified protein pockets. Bioassay‐guided fractionation, validated LC‐MS/MS identification and quantification, testing of authenticated compounds, direct enzyme and ion‐channel assays, pharmacokinetic characterization, and guideline‐compliant toxicological studies are required to bridge these evidence levels.

## 5. Limitations

This study is limited by the small pharmacological group size, absence of a prospective power calculation, incomplete records concerning randomization, allocation concealment, outcome‐assessor blinding, treatment formulation, carrageenan measurement instrumentation, and some extraction variables, as well as the absence of individual longitudinal records required for mixed‐effects analysis. Clear reporting of sample‐size justification, randomization, blinding, experimental procedures, and statistical methods constitutes a central component of the ARRIVE 2.0 recommendations [45].

The analytical limitations include incomplete HPLC‐PDA validation records, absence of LC‐MS/MS confirmation and validated standard‐based quantification, and chemical profiling of HEE rather than PF. Additional limitations include the absence of direct COX‐2, 5‐LOX, iNOS, or TRPA1 assays; a 72‐h tolerability screen restricted to HEE; computational rather than experimental ADMET assessment; rigid‐receptor docking with incomplete archived documentation of ligand microstates, cofactors, and metal treatment; and the inability to identify individual active compounds or infer clinical analgesic efficacy. These limitations materially constrain causal, mechanistic, safety, and translational interpretation.

## 6. Conclusion

The study provides exploratory evidence that *D. viscosa* HEE and PF can reduce nociceptive and inflammatory readouts in acute rodent models. The higher tested dose within each preparation generally produced a larger numerical effect, but the design does not establish a formal dose–response relationship or greater intrinsic potency of PF. The 72‐h tolerability finding applies only to HEE. HPLC‐PDA results support an HEE fingerprint with standard‐supported and putative annotations, while ADMET and docking identify potential preclinical candidate ligands for future testing rather than validated mechanisms. Confirmation requires complete analytical validation, chemical characterization of PF, raw‐data‐based repeated‐measures analysis, bioassay‐guided isolation, direct target assays, pharmacokinetics, and guideline‐compliant safety studies.

## Author Contributions

Sara Tlemcani, Abdessadek Essadek, and Chin‐Hung Lai conceived and designed the study. Sara Tlemcani, Amal Lahkimi, Anouar Hmamou, Amine Mounadi Idrissi, and Maryem Aichouch participated in plant preparation, extraction, phytochemical experiments, and data collection. Amal Elrherabi and Mohamed Bouhrim carried out the in vivo antinociceptive, anti‐inflammatory, and acute tolerability experiments and contributed to interpretation of the pharmacological results. Oussama Khibech and Abdessadek Essadek performed the in silico ADMET and molecular docking analyses and contributed to data analysis. Hicham Bekkari contributed to data interpretation and critically revised the manuscript. Sara Tlemcani, Oussama Khibech, and Abdessadek Essadek drafted the manuscript. Abdessadek Essadek and Chin‐Hung Lai supervised the work.

## Funding

This research was funded by Chung Shan Medical University under Project No. CSMU‐INT‐113‐18.

## Disclosure

All authors read and approved the final manuscript.

## Ethics Statement

All animal procedures were approved by the Institutional Ethics Committee for the Care and Use of Laboratory Animals, Faculty of Medicine and Pharmacy of Fez, Sidi Mohamed Ben Abdellah University, Fez, Morocco (approval no. 05/2023/LERHS). Experiments were conducted in accordance with relevant institutional, national, and international guidelines, with efforts to minimize animal suffering.

## Conflicts of Interest

The authors declare no conflicts of interest.

## Data Availability

The summary data reported in this article are included in the manuscript. Individual‐animal raw records were not available in the revision package used for the additional statistical reanalysis. Further information should be requested from the corresponding author.
